# Unveiling Co-Infection in Cystic Fibrosis Airways: Transcriptomic Analysis of *Pseudomonas aeruginosa* and *Staphylococcus aureus* Dual-Species Biofilms

**DOI:** 10.3389/fgene.2022.883199

**Published:** 2022-07-06

**Authors:** Andreia Patrícia Magalhães, Angela França, Maria Olívia Pereira, Nuno Cerca

**Affiliations:** ^1^ LIBRO—Laboratório de Investigação em Biofilmes Rosário Oliveira, Centre of Biological Engineering, University of Minho, Campus de Gualtar, Braga, Portugal; ^2^ LABBELS—Associate Laboratory, Braga, Portugal

**Keywords:** *Pseudomonas aeruginosa*, *Staphylococcus aureus*, RNA sequencing, dual-species biofilms, cystic fibrosis

## Introduction

Cystic fibrosis (CF) is a common heritable genetic disorder caused by a defect in the cystic fibrosis conductance regulator gene, resulting in several complications in the human body (Kreda et al., 2012). So far, the pathological changes in the lungs are best studied due to the high mortality rates linked to poor lung function and the recurrent development of severe biofilm-related infections (Flume et al., 2009; Ciofu et al., 2015). *Staphylococcus aureus* and *Pseudomonas aeruginosa* are the most prevalent pathogens that colonize structurally abnormal airways such as those diagnosed with CF and other chronic obstructive lung diseases (Lyczak et al., 2002; Hubert et al., 2013).

Although these bacteria seem to succeed with one another, CF patients acquire coinciding *P. aeruginosa* and *S. aureus* pulmonary infections, being co-infection usually associated with decreased lung function and increased frequency of pulmonary exacerbations (Limoli et al., 2016). Furthermore, *P. aeruginosa* and *S. aureus* pathogens adopt a biofilm mode of growth, which contributes to high tolerance to antibiotic treatment (Schobert and Jahn, 2010) and the recalcitrant nature of these chronic co-infections (Burmølle et al., 2006; Lopes et al., 2012), leading to significant patient morbidity and mortality (Cox et al., 2010). Interactions between *P. aeruginosa* and *S. aureus* have been widely studied, and it is commonly admitted that *P. aeruginosa* outcompetes *S. aureus*, perhaps outcompeting *S. aureus* for limited nutrients (Mashburn et al., 2005) or producing anti-staphylococcal compounds (DeLeon et al., 2014; Fugère et al., 2014), having *S. aureus* a minimal contribution to the overall course of the CF-associated biofilm infections (Bragonzi et al., 2012; Filkins et al., 2015). However, *P. aeruginosa* and *S. aureus* have been identified in the same lobe of CF lungs (Hogan et al., 2016; Wakeman et al., 2016) and are frequently diagnosed (Limoli et al., 2016; Zolin et al., 2019) as co-infecting species in CF patients. Moreover, *P. aeruginosa* strains isolated from early infection outcompete *S. aureus*, while strains isolated from chronic infection are less aggressive and can be co-cultivated with *S. aureus* (Frydenlund Michelsen et al., 2016; Limoli et al., 2017)*,* suggesting that these pathogens can interact *in vivo*.

In a previous study, we showed that *S. aureus* can grow and coexist with *P. aeruginosa* under dual-species biofilm conditions (Magalhães et al., 2021). Following up on these findings, and acknowledging that the molecular mechanisms behind these interactions are largely unknown, the purpose of the present study was, therefore, to identify the major transcriptomic features of *P. aeruginosa–S. aureus* dual-species biofilms, using high-throughput RNA-sequencing (RNA-seq). Herein, we described the full transcriptome of *P. aeruginosa* and *S. aureus* single- and dual-species biofilms and used a data analysis approach based on direct and functional gene interactions, namely gene set enrichment. These results will be invaluable for future functional studies involving *P. aeruginosa–S. aureus* interactions.

## Materials and Methods

### Bacterial Strains and Growth Conditions

*P. aeruginosa* UCBPP-PA14 and *S. aureus* ATCC 25923 were used throughout this work. Both bacteria were stored at −80 ± 2°C in tryptic soy broth (TSB, Liofilchem, Italy) supplemented with 20% glycerol. Before each assay, bacteria were sub-cultured from frozen stock preparations onto plates of TSB supplemented with 2% (w/v) agar and incubated aerobically at 37°C for 24 h.

Single- and dual-species biofilms formed by *P. aeruginosa* and *S. aureus* were prepared as described previously (Magalhães et al., 2017), with minor modifications. Briefly, overnight cultures of each species, grown in TSB at 37°C and 120 rpm in air conditions, were washed in sterile water and diluted in TSB to obtain 1 × 10^7^ CFU/ml. Bacterial numbers were estimated using optical density at 620 nm. Calibrations were previously performed for each bacterial strain to correlate the absorbance at 620 nm with the number of colony-forming units (CFUs) (Magalhães et al., 2021). For dual-species cultures, the suspended inoculum of each species was combined in a 1:1 ratio. Bacterial suspensions were dispensed in 24-well polystyrene plates (Orange Scientific, Braine-l`Alleud, Belgium) and incubated at 37°C on a horizontal shaker (120 rpm) for 24 h. Twenty-four-hour biofilms were then washed once with 0.9% NaCl, scraped from the bottom and the wall of the plates in 1 ml of RNA protect bacteria reagent (QIAGEN), which was diluted 2:1 in RNase-free water, as indicated by the manufacturer. After 5 min of incubation at room temperature, biofilm cells were harvested by centrifugation (20 min, 3,132×g) and RNA isolation was then performed. This assay was repeated six independent times.

### RNA Isolation and Library Construction

Total RNA was extracted using the RNeasy mini kit (QIAGEN) as optimized before (França et al., 2012). In brief, cells were suspended in 600 µl of the lysis buffer provided by the kit, plus 500 µl of phenol and 12 µl of β-mercaptoethanol. This suspension was transferred to a safe lock tube (2 ml) with 0.4 g of acid-washed 150–212 mm glass beads (Sigma) and using a BeadBug™ 6 (Benchmark Scientific) cell disruptor, the cells were lysed (4 × 4,500 rpm for 35 s, with 5 min intervals on ice between cycles). Finally, the tubes were centrifuged, and the suspension was transferred into a new tube. An equal volume of 70% of ethanol was added, the suspension was transferred into the RNeasy mini kit columns, and the manufacturer’s instructions were strictly followed. RNA quality was determined using the Agilent TapeStation 4200 (Agilent) and RNA quality indicators were above eight for all samples. Thereafter, total RNA obtained from three independent experiments was mixed and treated with TURBO DNase (Ambion) to degrade genomic DNA. Additionally, before the library construction, bacterial ribosomal RNA was removed using the NEBNext rRNA Depletion Kit (Bacteria). RNA libraries were prepared by strictly following the instructions of the kit KAPA HyperPrep (Roche). Libraries’ quality was determined using Agilent TapeStation 4200, and data were generated using Illumina NovaSeq 6000 from paired-end reads (2 × 150 bp).

### RNA-Seq Data Processing

After sequencing, Bcl2fastq version 2 (Illumina) was used for base calling and to convert the data to FASTQs files. CLC Genomics Workbench version 21 (QIAGEN) was then used for quality, ambiguity, and length trimming, using default settings. Thereafter, CLC was used for alignment using *S. aureus* (GenBank accession number: CP009361) and *P. aeruginosa* (GenBank accession number: NZ_CP034244) genomes, normalization of the reads (to transcripts per million—TPM), and for the analysis of differential gene expression (using single-species biofilms as control). Baggerley’s test (Pawitan et al., 2005) was applied to identify statistically significant alterations in single- vs. dual-species biofilms. Alterations with fold changes below two and *p*-values above 0.05 were discarded. Raw and analysed datasets have been deposited in NCBI’s Gene Expression Omnibus database and are accessible through GEO series accession number GSE195909.

### Functional Annotation

Gene function was annotated based on the Search Tool for the Retrieval of Interacting Genes/Proteins (STRING, version 11.5) (Szklarczyk et al., 2021), BLAST, Clusters of Orthologous Groups of proteins (COGs) (Tatusov et al., 2000; Galperin et al., 2015), Gene Ontology (GO) (Ashburner et al., 2000; Carbon et al., 2021), and Kyoto Encyclopedia of Genes and Genomes (KEGG) (Kanehisa and Goto, 2000; Kanehisa, 2019; Kanehisa et al., 2021) databases. The functional annotations were all determined based on the highest sequence similarity in these databases. GO enrichment analysis of differentially expressed genes was performed using standard GO terms from the Gene Ontology Resource and a Fisher’s exact test with FDR *p*-value <0.05 to estimate the statistical significance of the enrichment. Similarly, KEGG pathway analysis of differentially expressed genes was performed using KOBAS v2.1.1 (*p* < 0.05, hypergeometric test/Fisher’s exact test with FDR) (Bu et al., 2021).

## Data Description

### Analysis of Gene Expression

To study the responses of *P. aeruginosa* and *S. aureus* biofilm cells during interspecies interaction, we compared the gene expression profiles of both bacteria after 24 h of dual-species versus single-species growth. Earlier studies by Magalhães et al. (2021) have shown that *S. aureus* is present at high cell numbers in the *P. aeruginosa-*dominated 24 h dual-species biofilm consortia, indicating that the effects of interspecies interactions had not translated into significant changes in the population dynamics. Despite the evident coexistence interaction displayed after 24 h of co-culture, the transcriptome in each bacterium was affected by the presence of the other one when compared to the single-species transcriptome.

The total number of sequencing reads obtained ranged between 79,325,532 and 139,925,400 (Supplementary Table S1). A principal component analysis (PCA) of all samples showed a clear separation between the conditions under study (Supplementary Figure S1). Furthermore, heat maps revealed marked differences in the expression profile of either *P. aeruginosa* or *S. aureus* when grown as single- or dual-species biofilms (Supplementary Figure S2).

In the differential gene expression analysis of our RNA-seq data, single-species biofilms were used as the control, so the genes upregulated in *P. aeruginosa* could be interpreted as those positively regulated by *S. aureus*, whereas genes downregulated in *P. aeruginosa* would represent those negatively regulated by *S. aureus*, and vice-versa. We identified a total of 262 (6 upregulated and 246 downregulated) and 1,905 (101 upregulated and 1,804 downregulated) genes differentially expressed (fold-change ≥ 2 and *p* < 0.05) by *P. aeruginosa* and *S. aureus*, respectively (Supplementary Figure S3). The list of the 10 most highly up and downregulated genes in *P. aeruginosa* and *S. aureus* dual-species biofilms, as well as their annotated functions and COG families, are shown in Table 1.

**TABLE 1:** List of the 10 genes with higher fold-change among the differentially expressed genes (fold-change ≥ 2, and *p* < 0.05) in *P. aeruginosa* and *S. aureus* biofilms cultured under single- vs. dual-species conditions. COGs, Clusters of Orthologous Groups of proteins.

Gene	Annotation	COG category	Fold change (single- vs. dual-species biofilms)	*p*-value
Upregulated *S. aureus* genes				
KQ76_05755	Aspartate carbamoyltransferase	[F] Nucleotide transport and metabolism	478.97	1.37E-06
KQ76_10310	Hypothetical protein	[S] Function unknown	258.36	1.73E-02
KQ76_12410	PTS system trehalose-specific transporter	[G] Carbohydrate transport and metabolism	241.41	1.68E-03
KQ76_12710	Hypothetical protein	[S] Function unknown	179.40	6.13E-02
KQ76_13850	Mannose-6-phosphate isomerase	[G] Carbohydrate transport and metabolism	118.54	8.20E-04
KQ76_13345	Membrane protein	[R] General functional prediction only	95.65	1.08E-02
KQ76_13845	PTS mannose transporter subunit IIABC	[G] Carbohydrate transport and metabolism	78.44	0.00
KQ76_11410	6-Phospho-beta-galactosidase	[G] Carbohydrate transport and metabolism	48.05	6.29E-09
KQ76_13300	Hypothetical protein	[S] Function unknown	45.09	8.44E-12
KQ76_13275	PTS system glucose-specific transporter subunit IICBA	[G] Carbohydrate transport and metabolism	35.51	0.00
Downregulated *S. aureus* genes				
KQ76_05130	Hypothetical protein	[S] Function unknown	−2382.44	3.90E-20
KQ76_02615	50S ribosomal protein L1	[J] Translation, ribosomal structure, and biogenesis	−1505.05	4.37E-17
KQ76_05020	Chitinase	[F] Nucleotide transport and metabolism	−1366.48	0.00
KQ76_05285	Hypothetical protein	[S] Function unknown	−1314.97	0.00
KQ76_04050	Thioredoxin	[O] Posttranslational modification, protein turnover, and chaperones	−1257.63	1.09E-18
KQ76_05655	ftsL	[D] Cell cycle control, cell division, and chromosome partitioning	−1146.24	5.65E-18
KQ76_08480	Rrf2 family transcriptional regulator	[K] Transcription	−1023.22	4.45E-19
KQ76_08075	Hypothetical protein	[H] Coenzyme transport and metabolism	−921.95	1.32E-93
KQ76_06930	Hypothetical protein	[S] Function unknown	−889.90	5.56E-178
KQ76_08730	Translation initiation factor IF-3	[J] Translation, ribosomal structure, and biogenesis	−868.89	3.32E-165
Upregulated *P. aeruginosa* genes				
glcE	Glycolate oxidase subunit GlcE	[C] Energy production and conversion	22.13	3.84E-02
glcD	Glycolate oxidase subunit GlcD	[C] Energy production and conversion	17.60	3.92E-03
EIP97_RS04295	Transcriptional regulator AcoR	[K] Transcription and [Q] secondary metabolites biosynthesis and transport	3.29	1.23E-04
lldA	L-Lactate dehydrogenase LldA	[C] Energy production and conversion	3.26	4.34E-02
EIP97_RS23590	DUF3613 domain-containing protein	No category	2.26	2.69E-03
EIP97_RS04325	Hypothetical protein	No category	2.02	2.36E-02
Downregulated *P. aeruginosa* genes				
EIP97_RS14565	NADP-dependent glyceraldehyde-3-phosphate dehydrogenase	[C] Energy production and conversion	−22.54	3.69E-02
EIP97_RS14860	D-Glycerate dehydrogenase	[C] Energy production and conversion, [H] coenzyme transport and metabolism, and [R] general functional prediction only	−18.57	9.77E-03
EIP97_RS14875	TIM barrel protein	[G] Carbohydrate transport and metabolism	−15.90	4.66E-03
EIP97_RS14865	MFS transporter	[G] Carbohydrate transport and metabolism	−12.40	9.09E-03
pgl	6-Phosphogluconolactonase	[G] Carbohydrate transport and metabolism	−9.97	3.33E-02
EIP97_RS14870	Sugar kinase	[G] Carbohydrate transport and metabolism	−9.87	1.64E-02
pfkB	1-Phosphofructokinase	[G] Carbohydrate transport and metabolism	−9.69	0.00
EIP97_RS07570	PTS fructose-like transporter subunit IIB	[G] Carbohydrate transport and metabolism	−9.50	0.00
ptsP_3	Phosphoenolpyruvate–protein phosphotransferase	[G] Carbohydrate transport and metabolism and [T] signal transduction mechanisms	−9,30	0.00
edd	Phosphogluconate dehydratase	[E] Amino acid transport and metabolism and [G] carbohydrate transport and metabolism	−8.93	3.09E-02

**FIGURE 1:**
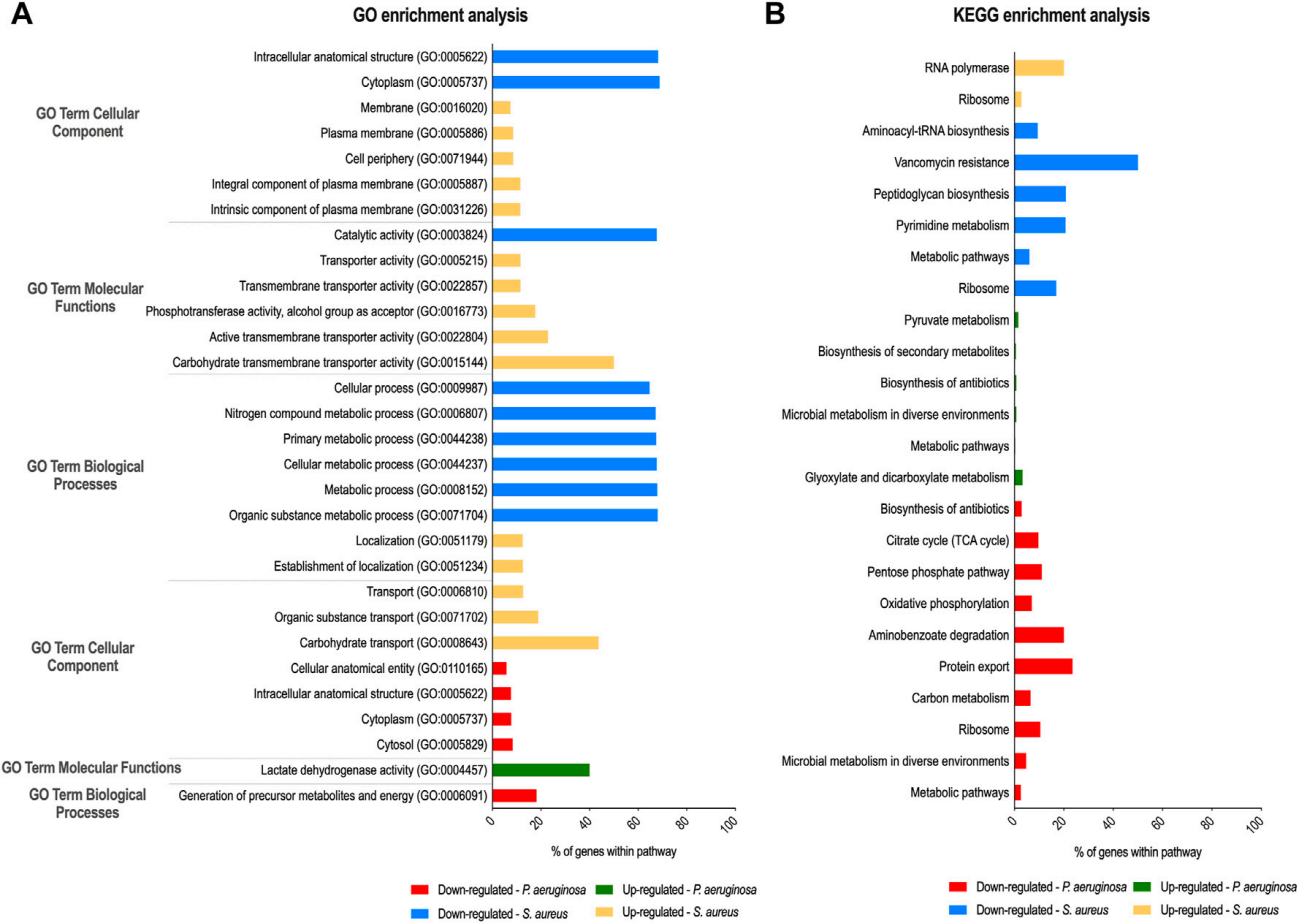
Gene ontology (GO) and Kyoto Encyclopedia of Genes and Genomes (KEGG) enrichment analysis of *P. aeruginosa* and *S. aureus* after dual-species biofilm growth. GO **(A)** and KEGG pathways **(B)** analyses were performed to identify, respectively, biological processes and pathways significantly enriched within differentially expressed genes in *S. aureus* and *P. aeruginosa*.

GO biologic process (including the three main categories: cellular component, molecular function, and biological process) and KEGG pathway analyses were performed on this cohort of genes (fold-change ≥ 2 and *p* < 0.05), and the GO terms and pathways enriched are reported in Figure 1. For *S. aureus* genes, in the biological process category, GO terms associated with metabolism were found significantly enriched among the downregulated genes. In the cellular component, “cytoplasm” and “intracellular anatomical structure” were the two enriched categories. In the molecular function, “catalytic activity” was the dominant category. Among the upregulated genes, “carbohydrate transmembrane transporter activity” in molecular function, “intrinsic component of plasma membrane” and “integral component of plasma membrane” in the cellular component, and “carbohydrate transport” in the biological process were the most enriched GO terms. As could be expected, many of the most significantly upregulated *S. aureus* genes listed in Table 1 have functions related to these pathways. Regarding *P. aeruginosa* GO analysis, only six different subcategories were found to be significantly enriched (Figure 1A). Among these subcategories, “cytosol”, “cytoplasm,” and “intracellular anatomical structure” in the cellular component and “generation of precursor metabolites and energy” for biological processes were those receiving the most abundant annotations for *P. aeruginosa* downregulated genes. “Lactate dehydrogenase activity” was the only GO term significantly enriched for *P. aeruginosa* upregulated genes in the molecular function category. In addition, the two annotated genes (*glcE* and *lldA*) of this pathway comprised the *P. aeruginosa* top 10 upregulated genes shown in Table 1. In the KEGG annotations, significantly expressed genes were divided into 24 subcategories, with major alterations occurring in the downregulated genes of both species (Figure 1B). Among the downregulated genes in the *S. aureus* group, the enriched KEGG pathways were related to “vancomycin resistance,” followed by “peptidoglycan biosynthesis” and “pyrimidine metabolism.” Within the group of DOWNREGULATED genes in *P. aeruginosa* “protein export” and “aminobenzoate degradation” pathways were the most enriched pathways. The COG analysis showed that the majority of the differentially expressed genes have no assigned category based on the categories of Clusters of Orthologous Groups (COGs) (Supplementary Figure S3). However, there is also a high number of downregulated genes differentially expressed in *P. aeruginosa* that belong to energy production and conversion (category C; 32 genes) and, in the case of *S. aureus*, genes that belong to the amino acid transport and metabolism (category E; 163 genes)*.*

Our dual-transcriptome analysis of *P. aeruginosa* and *S. aureus* reveals that the adaptations are dominated by metabolic changes since the largest number of differentially expressed genes belongs to the functional classes “metabolism” and “transport.” In particular, our data also confirmed previous observations that *P. aeruginosa* drives *S. aureus* into fermentation (Filkins et al., 2015; Tognon et al., 2019). The lactate dehydrogenase (*ldh1* and KQ76_13385), L-lactate permease (KQ76_12340), and acetolactate synthase (KQ76_11515) genes were upregulated 2- to 11-fold in the dual-species biofilms, indicating that *S. aureus* preferentially converted pyruvate into lactate. In line with these findings, one of the most upregulated genes of *P. aeruginosa* in response to *S. aureus* was the membrane-bound L-lactate dehydrogenase lldA (3-fold increase), suggesting that *P. aeruginosa* takes up lactate secreted by *S. aureus* to use it as a carbon and energy source. A study performed by Camus et al. (2020) suggests that acetoin may also play a role in metabolic interactions between *P. aeruginosa* and *S. aureus. P. aeruginosa* demonstrated an enhanced ability to catabolize acetoin produced by *S. aureus* as an alternative carbon source, resulting in increased survival during co-culture and avoiding the toxic accumulation of acetoin on *S. aureus* (Camus et al., 2020). Interestingly, we observed that the gene encoding the transcriptional regulator AcoR (EIP97_RS04295), described to be responsible for acetoin catabolism (Liu et al., 2018), was 3-fold upregulated in *P. aeruginosa* dual-species biofilms (Table 1). Moreover, we also found no evidence for the induction of anti-staphylococcal molecules in *P. aeruginosa*, suggesting that no direct competition prevails during dual-species biofilm growth. Additionally, a relatively low number of genes were significantly differentially expressed in the *P. aeruginosa* dual-species biofilm when compared to *S. aureus,* which had a more marked transcriptomic response, further suggesting that this species is less affected by *S. aureus* as we have phenotypically shown earlier (Magalhães et al., 2021). A similar trend was observed in other studies comparing mono- and co-cultures of *P. aeruginosa* and *S. aureus* (Filkins et al., 2015; Miller et al., 2017), indicating that *P. aeruginosa* appears to easily maintain itself as a dominant organism in various *in vitro* systems.

Overall, these data enabled us to identify key pathways and genes involved in the interaction of both bacteria during dual-species biofilm growth that warrant further investigations. Hence, these results may help unveil key molecular mechanisms driving the coexistence of these pathogens that might impact infection progression and the selection of potential targets for future studies aiming to develop preventive and/or therapeutic strategies for *P. aeruginosa–S. aureus* biofilm infections in CF, as well as in other diseases involving co-infection with these pathogens.

## Data Availability

The datasets presented in this study can be found in online repositories. The names of the repository/repositories and accession number(s) can be found in the article.
