# Supplementary material for: Unveiling Co-Infection in Cystic Fibrosis Airways: Transcriptomic Analysis of Pseudomonas aeruginosa and Staphylococcus aureus Dual-Species Biofilms
Source: Front Genet. 2022 Jul 6;13:883199. doi: 10.3389/fgene.2022.883199 (PMC9298864; doi:10.3389/fgene.2022.883199)
Supplement: Supplementary file 1 [file DataSheet1.docx]

Supplementary Material

**Unveiling co-infection in cystic fibrosis airways: Transcriptomic analysis of *Pseudomonas aeruginosa* and *Staphylococcus aureus* dual-species biofilms**

Andreia Patrícia Magalhães^1,2^, Angela França^1,2*^, Maria Olívia Pereira^1,2^, Nuno Cerca^1,2^

^1^ Centre of Biological Engineering, LIBRO – Laboratório de Investigação em Biofilmes Rosário Oliveira, University of Minho, Campus de Gualtar, 4710-057 Braga, Portugal

^2^ LABBELS –Associate Laboratory, Braga, Guimarães, Portugal

*** Correspondence:** Angela França, [afranca@ceb.uminho.pt](mailto:afranca@ceb.uminho.pt)

**Running Title:** Transcriptome of *P.aeruginosa*/*S.aureus* biofilms

**Supplementary Table 1.** Summary of the sequencing results. S, single; M, mixed

| **Sample Identity** | **Sample type** | **Read type** | **Total count Reads** | **Reads mapped in pairs** | **Reads mapped in broken pairs** |
| --- | --- | --- | --- | --- | --- |
| Pa_S_1_ | *P. aeruginosa* | 150 × 2 paired-end | 133,397,784 | 128,429,678 | 2,656,657 |
| Pa_S_2_ | *P. aeruginosa* | 150 × 2 paired-end | 109,742,548 | 100,748,864 | 2,915,604 |
| Pa_M_1_ | *P. aeruginosa+S. aureus^*1^* | 150 × 2 paired-end | 134,531,674 | 127,208,514 | 3,110,422 |
| Pa_M_2_ | *P. aeruginosa+S. aureus^*1^* | 150 × 2 paired-end | 139,925,400 | 130,754,942 | 6,192,986 |
| Sa_S_1_ | *S. aureus* | 150 × 2 paired-end | 79,384,900 | 76,685,234 | 1,186,500 |
| Sa_S_2_ | *S. aureus* | 150 × 2 paired-end | 79,325,532 | 76,919,300 | 1,100,071 |
| Sa_M_1_ | *P. aeruginosa+S. aureus^*2^* | 150 × 2 paired-end | 134,531,674 | 212,054 | 13,540 |
| Sa_M_2_ | *P. aeruginosa+S. aureus^*2^* | 150 × 2 paired-end | 139,925,400 | 520,816 | 32,570 |

^*1^RNA-Seq reads alignment was performed using only the *P. aeruginosa* genome.

^*2^RNA-Seq reads alignment was performed using only the *S. aureus* genome.


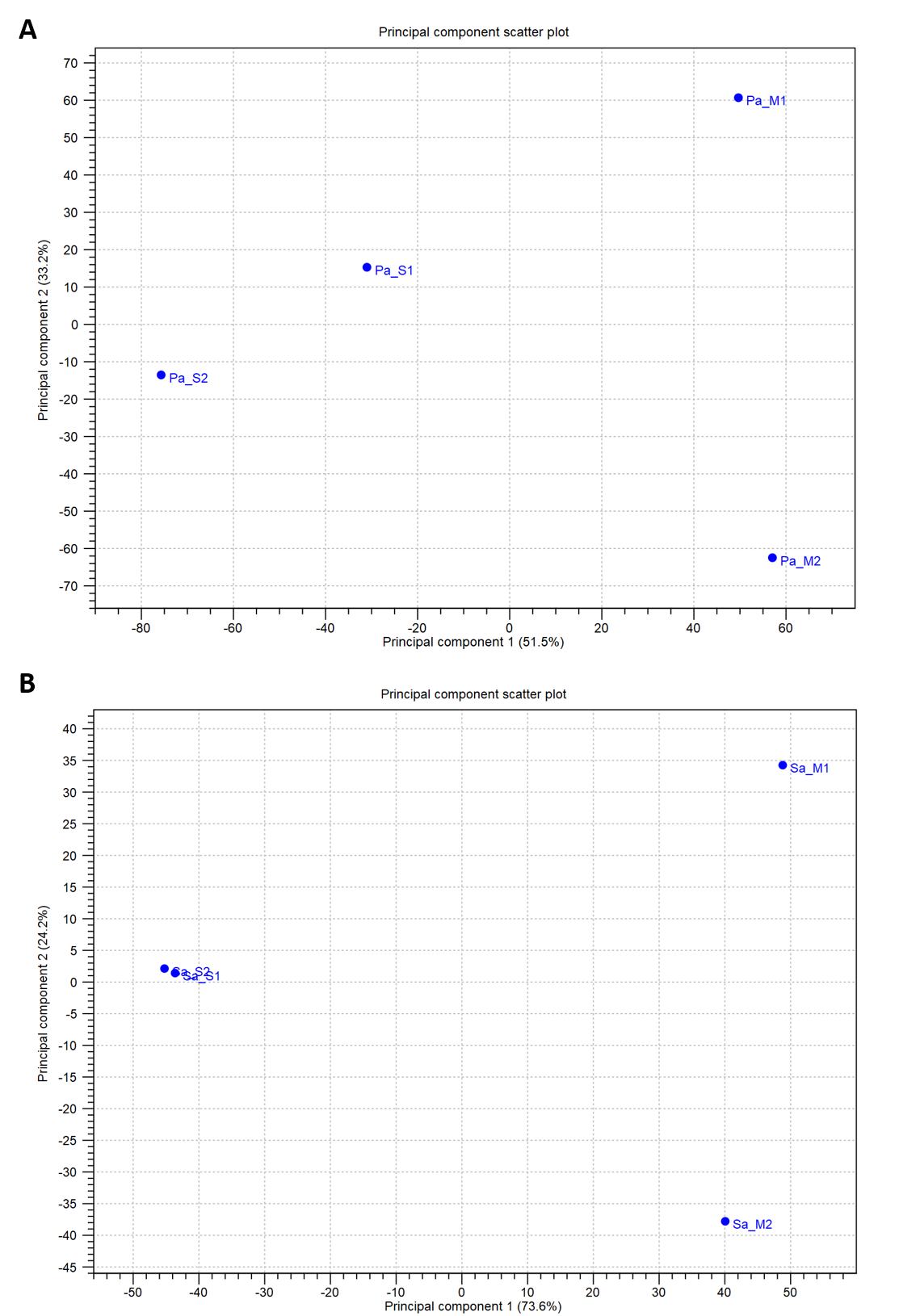


**Supplementary Figure 1.** Principal component analysis (PCA) of each biological replicate of **(A)** *P. aeruginosa* (Pa) *and* **(B)** *S. aureus* (Sa) cultured under single- (S1 and S2) and dual-species (M1 and M2). PCA was performed using CLC genomics workbench version 21.


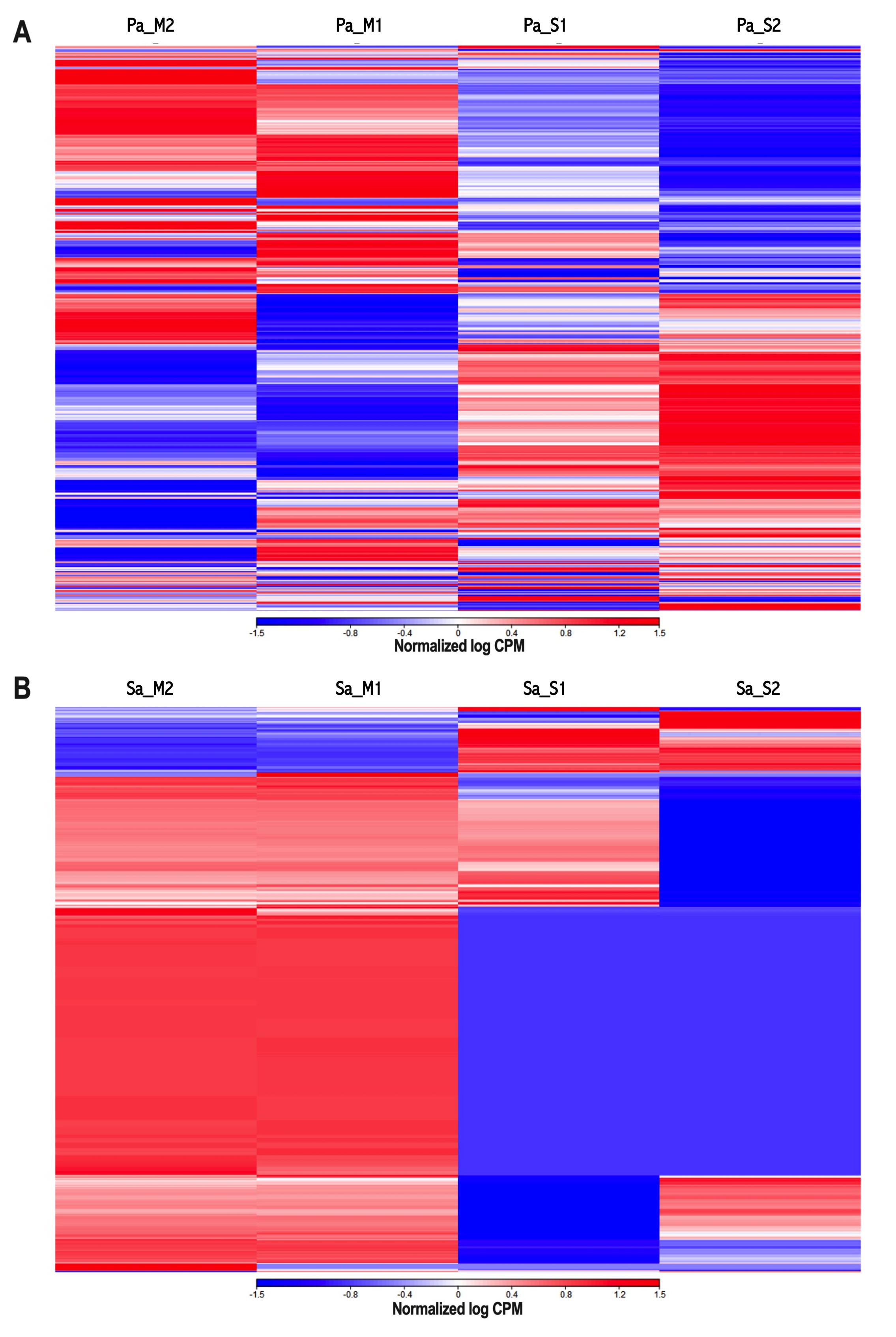


**Supplementary Figure 2.** Heat map of each biological replicate showing the gene expression pattern of *P. aeruginosa* (Pa) **(A)** and *S. aureus* (Sa) **(B)** samples in single- (S1 and S2) and dual-species (M1 and M2) growth conditions. Heat maps were performed using CLC genomics workbench version 21. CPM, counts per million.


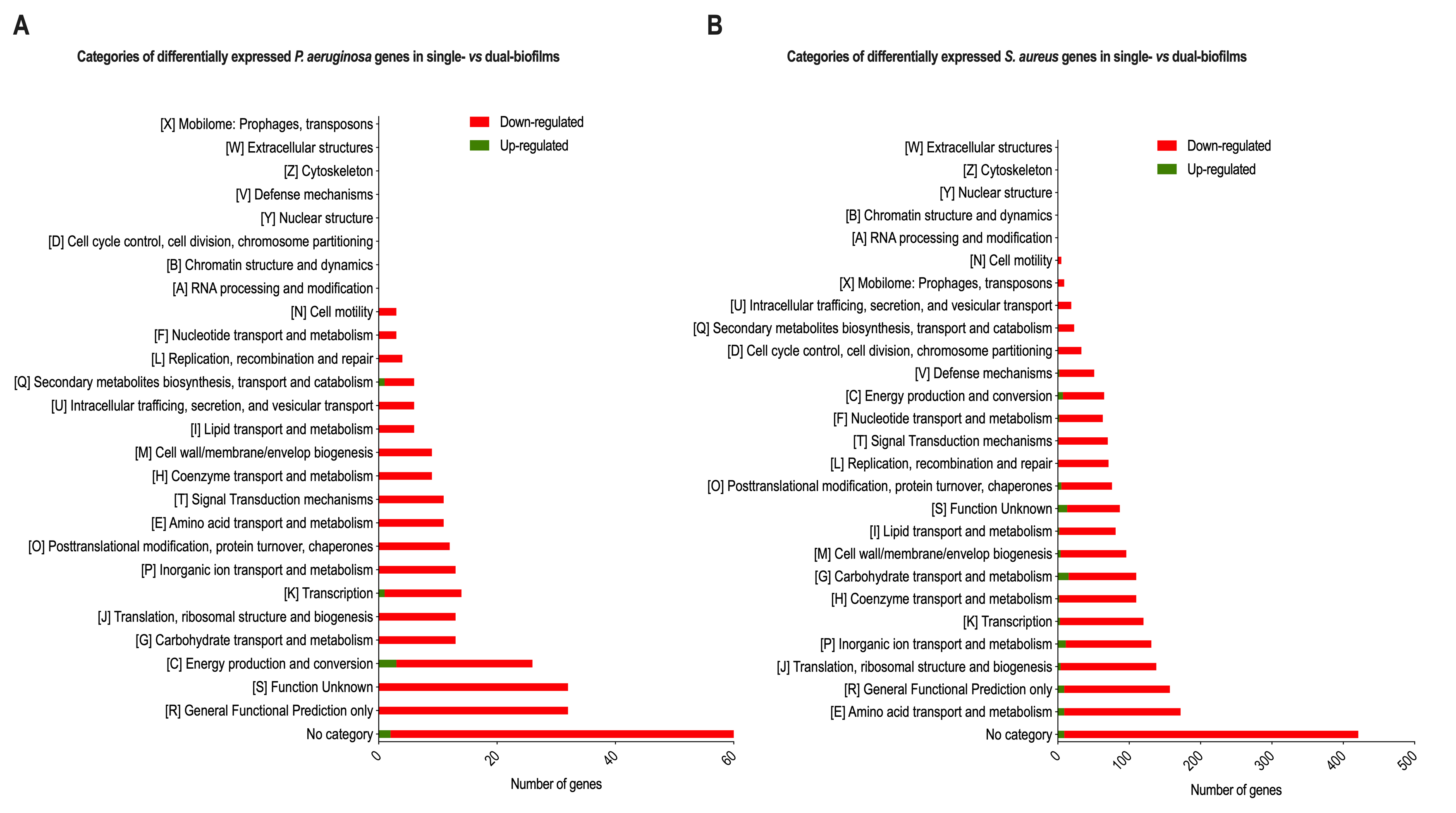
**Supplementary Figure 3.** The number of up- and downregulated genes per COG category from *P. aeruginosa* **(A)** and *S. aureus* **(B)** samples. Differentially expressed genes (fold-change ≥ 2 and p < 0.05) are relative to single- *versus* dual-species biofilms.
